# NGLY1 deficiency: estimated incidence, clinical features, and genotypic spectrum from the NGLY1 Registry

**DOI:** 10.1186/s13023-022-02592-3

**Published:** 2022-12-17

**Authors:** Caroline R. Stanclift, Selina S. Dwight, Kevin Lee, Quirine L. Eijkenboom, Matt Wilsey, Kristen Wilsey, Erica Sanford Kobayashi, Sandra Tong, Matthew N. Bainbridge

**Affiliations:** 1 Grace Science Foundation, P.O. Box 114, Menlo Park, CA USA; 2 Rady Children’s Institute for Genomic Medicine, 3020 Children’s Way, San Diego, CA USA

**Keywords:** NGLY1 deficiency, Congenital disorder of deglycosylation, Incidence, Rare diseases, Patient registry

## Abstract

**Purpose:**

NGLY1 Deficiency is an ultra-rare, multisystemic disease caused by biallelic pathogenic *NGLY1* variants. The aims of this study were to (1) characterize the variants and clinical features of the largest cohort of NGLY1 Deficiency patients reported to date, and (2) estimate the incidence of this disorder.

**Methods:**

The Grace Science Foundation collected genotypic data from 74 NGLY1 Deficiency patients, of which 37 also provided phenotypic data. We analyzed *NGLY1* variants and clinical features and estimated NGLY1 disease incidence in the United States (U.S.).

**Results:**

Analysis of patient genotypes, including 10 previously unreported *NGLY1* variants, showed strong statistical enrichment for missense variants in the transglutaminase-like domain of *NGLY1* (*p* < 1.96E−11). Caregivers reported global developmental delay, movement disorder, and alacrima in over 85% of patients. Some phenotypic differences were noted between males and females. Regression was reported for all patients over 14 years old by their caregivers. The calculated U.S. incidence of NGLY1 Deficiency was ~ 12 individuals born per year.

**Conclusion:**

The estimated U.S. incidence of NGLY1 indicates the disease may be more common than the number of patients reported in the literature suggests. Given the low frequency of most variants and proportion of compound heterozygotes, genotype/phenotype correlations were not distinguishable.

**Supplementary Information:**

The online version contains supplementary material available at 10.1186/s13023-022-02592-3.

## Introduction

*N*-glycanase 1 (NGLY1) Deficiency (MIM #615273) is an ultra-rare, autosomal recessive disorder caused by loss of function variants in *NGLY1* [1]. The disorder is characterized by five core features: (1) global developmental delay and/or intellectual disability, (2) a primarily hyperkinetic movement disorder (3) transient elevation of liver transaminases (4) hypo- or alacrima and (5) peripheral neuropathy [1–3]. Additional signs and symptoms of the disease may include epilepsy, feeding difficulties, failure to thrive, hypotonia, central and/or obstructive sleep apnea, microcephaly, non-specific findings on brain MRI (delayed myelination; small corpus callosum, anterior commissure, and/or cerebellar atrophy; prominent cisterna magna and/or enlarged ventricles) [3], and orthopedic complications such as progressive scoliosis and joint contractures [4]. Caregivers commonly report regression of motor and/or cognitive function [2]. As NGLY1 Deficiency presents with a complex neurologic phenotype, the differential diagnosis is broad and may include congenital disorders of glycosylation (CDG), mitochondrial disorders, MECP2-related disorders, and neurotransmitter disorders. Patients may be given a non-specific diagnosis of cerebral palsy. The constellation of symptoms and the range of severity varies between patients, making diagnosis and prognosis challenging [2, 3].

*NGLY1* encodes the enzyme *N*-glycanase 1 (NGLY1). This 654 amino acid long protein consists of three major domains: the N-terminus PUB domain (present in PNGase/UBA or UBX-containing proteins and known to bind VCP, the C-terminus PAW domain (a high mannose-type glycan-binding domain that recognizes oligosaccharide substrates), and the transglutaminase-like domain (enzymatic domain) in the center of the protein. The enzymatic domain can be further subdivided into the transglutaminase core and the Rad4 transglutaminase-like domain. NGLY1 cleaves *N*-glycans from other proteins and is the only known cytosolic enzyme known to catalyze hydrolysis of the amide bond between an *N*-linked glycan and the asparagine of a protein [5]. As such, the underlying biology of NGLY1 Deficiency is distinct from that of congenital disorders of glycosyation (CDG) [6]. NGLY1 function is critical for the endoplasmic reticulum-associated protein degradation (ERAD) pathway that recognizes and eliminates unfolded or incorrectly processed glycoproteins [7]. Loss of NGLY1 function leads to accumulation of aspartylglucosamine (abbreviated GlcNAc-Asn or GNA) that can be detected in patient urine, plasma, and dried blood spots [5, 8]. In addition to affecting the ERAD pathway, loss of NGLY1 function impacts numerous cellular processes such as proteasomal homeostasis, mitophagy, BMP signaling, AMP kinase signaling, and ferroptosis, which may explain the multisystemic effects seen in patients with the disorder [6, 9]. Deglycosylation is critical for protein recycling and in vitro evidence suggests that misfolded products of the endoplasmic reticulum (ER) aggregate within the cells of NGLY1 patients and cause deleterious effects on the cytoplasm, ER, and mitochondria, leading to disease [10].

*NGLY1* is located on chromosome 3p24.2. To date, 46 pathogenic variants in *NGLY1* have been described in the literature [2]. Pathogenic variants include missense variants, truncations, small deletions, and splice-site variants. Truncating variants have been identified in each of the twelve exons of the gene [6]. With the exception of a slightly more common European allele, p.Arg401*, (MAF: 0.04% in gnomAD), the majority of known deleterious alleles are either extremely rare or have no known minor allele frequency.

Until recently, the largest cohorts of NGLY1 Deficiency patients included 8 patients from a retrospective chart review and 12 patients enrolled in an NIH study [1, 3]. Early findings from a subsequent prospective, longitudinal NGLY1 Deficiency Natural History Study (NCT03834987) reported on 29 patients [2]. Including those studies as well as case reports [11–28], there have been 65 unique NGLY1 Deficiency patients reported in literature. Due to the small number of patients described in the literature, the incidence of this disease was heretofore unknown. This paucity of information makes it challenging for clinicians to accurately provide disease counseling for parents and caregivers. Although NGLY1 Deficiency is well characterized, patients are often misdiagnosed or remain undiagnosed for years, likely due to clinician unfamiliarity with this ultra-rare disorder. As the use of next-generation sequencing technology increases, it is likely that new or previously undiagnosed patients will be identified. Here we present genotypic data for 74 patients identified by the Grace Science Foundation and caregiver-reported survey data for 37 of them, representing the largest NGLY1 Deficiency cohort to date. We describe the frequency of NGLY1 Deficiency symptoms for 11 previously unreported individuals, present 10 novel pathogenic *NGLY1* variants, and calculate an estimated incidence of the disease using a previously described method for estimating the incidence of rare diseases [29].

## Materials and methods

### Patient cohort

Individuals eligible to participate in the Grace Science Foundation NGLY1 Registry were enrolled as previously described [4]. Grace Science Foundation (https://gracescience.org/) is a 501c(3) research and patient advocacy organization dedicated to NGLY1 Deficiency. Institutional Review Board (IRB) approval for the NGLY1 Registry was obtained from Stanford University IRB (#41906) and later Genetic Alliance IRB (#GSF001).

In brief, genetic test reports and/or biochemical testing were collected and reviewed by a genetic counselor (C.R.S) to confirm biallelic pathogenic variants in *NGLY1*. Once confirmed, caregivers gave informed consent for their children or dependents to participate in the NGLY1 Registry and provided brief demographic information.

### Survey collection and analysis

Caregivers had the option to answer a questionnaire designed to capture medical history and development outcomes relevant to NGLY1 Deficiency (Additional file 1). Responses were source-verified with medical records when available. Data was compiled through secure information storage systems maintained by the Grace Science Foundation and shared in a deidentified manner with the investigator group. Patient-friendly descriptions used for NGLY1 Deficiency symptoms in the survey were mapped to relevant Human Phenotype Ontology (HPO) terms, a standardized vocabulary of phenotypic abnormalities encountered in human disease, for analysis. Quantitative analyses were conducted in Microsoft Excel or R Studio. Descriptive statistics were used to summarize the characteristics between male and female patients with NGLY1 Deficiency post hoc. *p* values < 0.05 were considered significant.

### Incidence calculation

Incidence for NGLY1 Deficiency was calculated as previously described [29]. Briefly, all variants from patients of European ancestry, including white Hispanic, collected by the Grace Science Foundation NGLY1 Registry were used for the calculation. Minor allele frequencies, where available, were derived from the maximum allele frequency of “Europeans” in gnomAD. The threshold of “rareness”, r, was set to 1 × 10^−4^. The estimated lambda was determined to be 0.567.

## Results

### Patient cohort demographics

As of January 2022, the Grace Science Foundation NGLY1 Registry included 62 unrelated individuals and six sibling pairs for a total of 74 NGLY1 Deficiency patients; 36 of these patients have not been previously reported (Additional file 1: Table S1).

**Table 1:** Demographic characteristics for patients enrolled in the NGLY1 patient registry for whom phenotypic information is available (N = 37), including age of symptom onset and age of diagnosis. Categorical variables are presented as number and percentage N (%). Quantitative variables are presented as mean and standard deviation, as well as median and range

	N = 37		N = 37
**Sex**		**Age at Enrollment**	
Male	19 (51%)	Mean (SD)	8.1 years (5.8)
Female	18 (49%)	Median (Min,Max)	7 years (0.24)
**Country of Origin**	**% of Cohort**	**Symptom Onset**	
Brazil	2 (5.4%)	Mean (SD)	4.5 months (4.7)
Canada	5 (13.5%)	Median (Min,Max)	4 months (0.24)
China	1 (2.7%)		
Denmark	1 (2.7%)	**Age at Diagnosis**	
France	3 (8.1%)	Mean (SD)	6.6 years (5.1)
Germany	1 (2.7%)	Median (Min,Max)	5 years (0.20)
India	1 (2.7%)		
Israel	1 (2.7%)		
Mexico	1 (2.7%)		
Spain	1 (2.7%)		
The Netherlands	1 (2.7%)		
USA	19 (51.4%)		

Phenotypic data was available for 37 of the 74 individuals (29 unrelated individuals and four sibling pairs) (Table 1). Unfortunately, phenotypic data was not collected from all participants due to an unexpected closure of the database registry. Of the 37 individuals, 19 were male and 18 were female. Age at time of survey ranged from 4 months to 24 years (mean 8.14 years). Individuals represented 12 countries (Brazil, Canada, China, Denmark, France, Germany, India, Israel, Mexico, Spain, the Netherlands, and the United States) and were predominantly of European descent (30 of 37).

### Caregiver reported NGLY1 deficiency-related symptoms

**Fig. 1:**
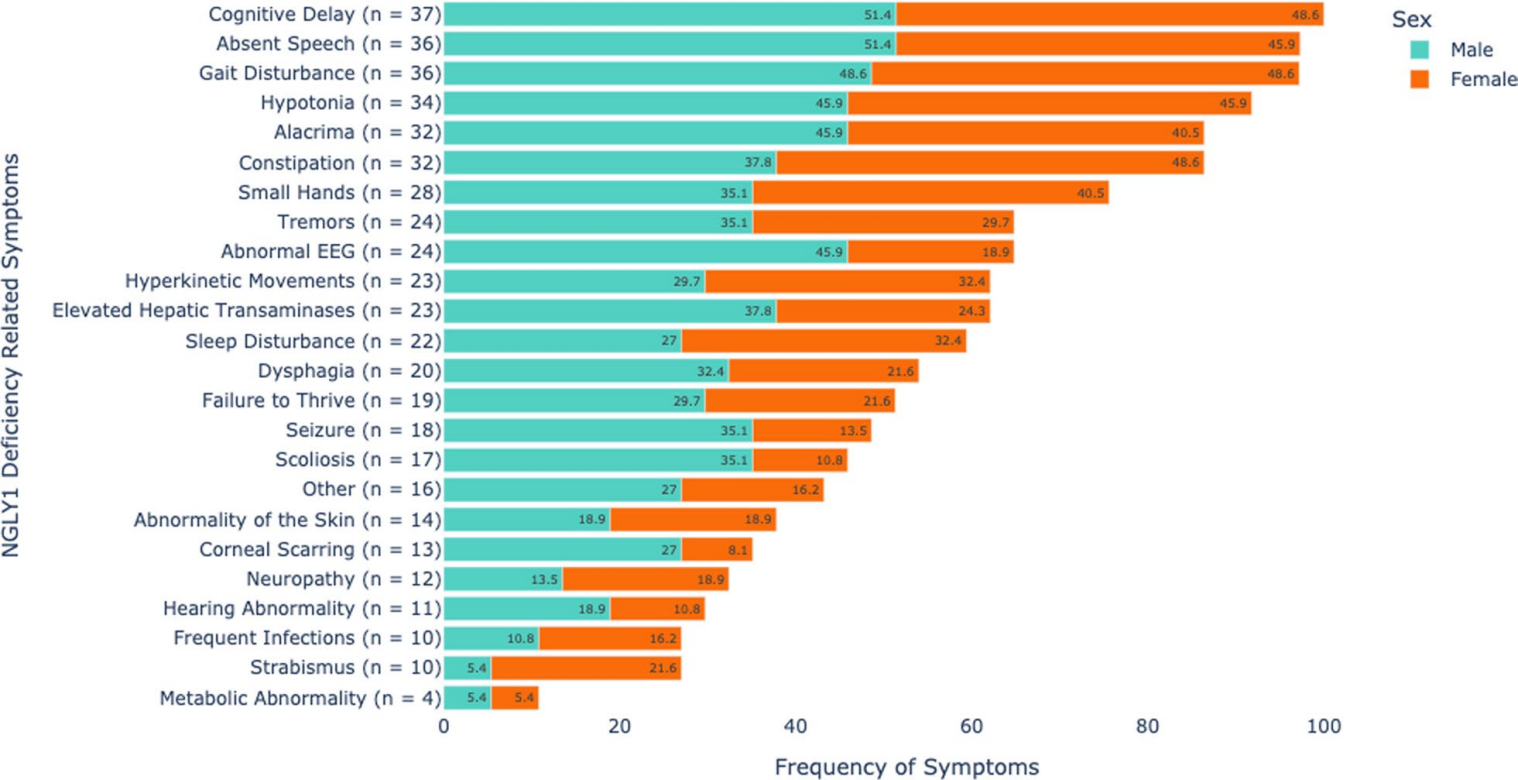
Frequency of NGLY1 Deficiency Related Symptoms by Caregiver Report. Frequency of symptoms reported in our NGLY1 Deficiency cohort for whom phenotypic data was available (N = 37; 19 males, 18 females). This bar chart demonstrates the number of patients affected by each clinical feature surveyed in the questionnaire. Males are colored in turquoise and females are colored in orange. The percentage represents the proportion of males or females with relevant symptoms compared to the whole cohort

Caregivers reported age of symptom onset ranging from prenatal to 24 months of age, with a median age of 4 months. The mean age of diagnosis was 6.6 years. A total of 23 specific symptoms were documented by caregivers (Fig. 1). Cognitive delay and absent speech were reported in 37 of 37 (100%) and 36 of 37 (97.3%) individuals in this cohort, respectively. Movement disorder symptoms, including gait disturbance (36 of 37; 97.3%) and hypotonia (34 of 37; 91.9%), were noted in the vast majority. Also commonly reported were alacrima and severe constipation: both were identified in 32 of 37 (86.5%) of individuals. Additional common neurologic features were tremors (24 of 37; 64.9%,), hyperkinetic movements (23 of 37; 62.2%), abnormal electroencephalogram (EEG) results (24 of 37; 64.9%), and seizures (18 of 37; 48.6%). Elevated hepatic transaminases were reported in 23 of 37 (62.2%) individuals. Failure to thrive was reported in 19 of 37 (51.4%) individuals. Results of additional symptoms surveyed included: sleep disturbance (22 of 37; 59.5%), dysphagia (20 of 37; 54.1%,), scoliosis (17 of 37; 45.9%), small hands and feet (28 of 37; 75.7%), abnormality of the skin (14 of 37; 37.8%), corneal scarring (13 of 37; 35.1%), neuropathy (12 of 37; 32.4%), hearing abnormality (11 of 37; 29.7%), frequent infections (10 of 37; 27%), strabismus (10 of 37; 27%), and metabolic abnormalities (4 of 37; 10.8%).

In total**,** 17 of 37 (46%) individuals in our cohort experienced motor and/or cognitive regression. Every patient 14 years or older was reported to have diminishing motor function. Loss of motor abilities coincided with onset of puberty for two female patients and after a surgery or hospitalization event for four patients. For one patient, motor and cognitive regression was noted at only two years of age.

Beyond the symptoms specifically surveyed, 16 of 37 (43.2%) patients were reported by their caregiver to experience additional clinical features. These included hypospadias, frequent pneumonia or airway obstruction, recurrent fractures secondary to osteopenia, laryngomalacia, Hirschsprung's disease, anxiety, eosinophilic esophagitis, microcephaly, skin hypo- or hyper-pigmentation, and exocrine pancreatic insufficiency. Multiple caregivers reported symptoms impacting the eyes and/or vision abnormalities.

Additionally, caregiver-reported data demonstrated statistically significant phenotypic differences between the sexes for some symptoms associated with NGLY1 Deficiency. Scoliosis was more frequently reported in males compared to females, with 13 of 19 (68.4%) males affected compared to 4 of 18 (22.2%) females (*p* = 0.016, Fisher’s exact). Abnormal EEG findings were reported in 17 of 19 (89.5%) males compared to 7 of 18 (38.9%) females (*p* = 0.004, Fisher’s exact). Other features approach but do not meet statistical significance. Seizures were reported in 13 of 19 (68.4%) males compared to 5 of 18 (27.8%) females. Additionally, males showed a higher frequency of corneal scarring compared to females; however, strabismus was reported in 8 of 18 (44.4%) females compared to 2 of 19 (10.5%) males. All females reported constipation compared to 14 of 19 (73.7%) males.

### Caregiver reported growth parameters

**Fig. 2:**
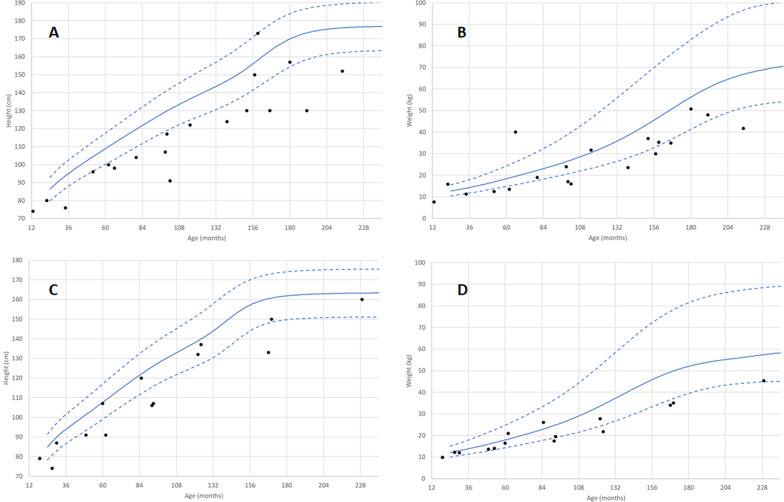
Height and Weight by Caregiver Report. Growth Curves. Height (panel **A**, males, **C**, females) and weight (panel **B**, males, **D** females) for each individual NGLY1 patient aged 2 to 20 years old compared to CDC child growth standards (50th-tile solid line, 3rd and 97th-tile dashed line). Each black dot represents a single measurement for one individual

Caregivers reported NGLY1 patients experienced poor growth with an average Z score of −2.6 and −1.4 for height and weight, respectively (Additional file 1: Table S2). Males were more likely to be underweight compared with females, with 9 of 17 (52.9%) of males measuring below the 3rd percentile for their age and sex compared to 4 of 14 (28.6%) of females (Fig. 2B, D). Males were more affected than females with respect to height, with 14 of 17 (82.4%) of males below 3rd percentile for their age and sex compared to only 6 of 13 (46%) of females (*p* = 0.05, Fisher’s exact) (Fig. 2A, B). In 7 individuals, growth was affected enough to necessitate placement of a G-tube to aid with weight gain.

### Characterization of pathogenic variants in *NGLY1*

**Fig. 3:**
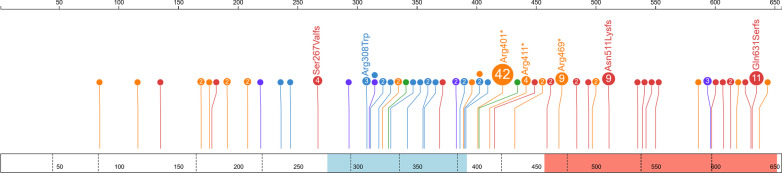
Lollipop Plot of Variants in *NGLY1*. 56 pathogenic variants and their frequency. Pathogenic variants (orange, nonsense; red, frameshift; purple, splice; green, indel; blue, missense) indicated by circles, with pathogenic variant count (n > 1) and pathogenic variant label (n > 2). Protein domains PAW (red) and transglutaminase-like (light blue), exon-exon boundaries (dotted) lines are shown across the protein (box). Amino acid count is given at top

Combining the 65 patients previously reported in the literature with the unique individuals in our registry cohort, we identified 103 NGLY1 Deficiency patients with a total of 56 pathogenic variants (Fig. 3). We found that truncating pathogenic variants in *NGLY1* occur throughout the length of the gene and the majority are predicted to result in nonsense mediated decay (NMD) [30]. Interestingly, a surprising number of truncating pathogenic variants occur in the last exon. Because these variants would not be predicted to undergo NMD, they may be indicative of the importance of the oligosaccharide binding PAW domain to NGLY1 ‘s function. In contrast to truncating pathogenic variants, the majority (89%) of pathogenic missense variants were found in the transglutaminase-like domain of *NGLY1*, which comprises only ~ 17.7% of the total protein length [31]. The strong statistical enrichment for missense pathogenic variants in this region (*p* < 1.96E−11; Binomial) is likely indicative of the evolutionary and functional importance of this domain and the relative tolerance of other regions of the protein to missense variants. The remaining missense pathogenic variants (p.Trp244Arg and p.Trp236Cys) are in close three-dimensional proximity (~ 3.3 Å) to p.Cys309 and may disrupt the orientation of the alpha-helix (residues 309–323) that forms part of the catalytic domain (residues 275–392) (Additional file 1: Fig. S1) [32]. This knowledge may be useful in informing variant classification for future novel missense variants as they are identified.

### Incidence of NGLY1 deficiency

NGLY1 Deficiency is an ultra-rare disease, which makes calculating the incidence challenging. Although most data collected in our registry includes individuals of European ancestry, other genetic ancestries are also represented. Notably, some of the minor allele frequencies (MAF) are highest in gnomAD’s East Asian population (Additional file 1: Table S3).

To estimate incidence, we used a previously described method [29], which attempts to consider not only the MAF of observed alleles but also to estimate the total MAF of all other alleles, including pathogenic variants yet to be observed. To maximize power for our calculation we chose to concentrate on individuals of European ancestry. We calculated that as many as 58.3% of the causative alleles for NGLY1 Deficiency have yet to be observed, due to the very large number of pathogenic variants in the registry that have no MAF in gnomAD. This suggests a relatively long-tailed Poisson distribution of allele frequencies (Additional file 1: Fig. S2) and that the total MAF of unobserved alleles is equal to 2.59 × 10^−4^ and the total MAF is equal to 1.85 × 10^−3^. As there are approximately 3.8 million births in the United States annually, provided this MAF calculation holds for the majority of the US population, our calculations would suggest that on average 12.4 children are born each year in the United States with this disease.

### Lifespan

Of the 103 patients identified via our patient advocacy organization as of January 2022 and reported in the literature (Additional file 1: Fig. S3), 16 (15.5%) individuals are currently known to be deceased (Additional file 1: Table S1). The mean and median ages of death for those 16 individuals were 11.9 and 13 years old, respectively (range 6 months to 22 years old). Causes of death included respiratory failure, adrenal insufficiency and, in a single instance, cardiac arrest.

## Discussion

NGLY1 Deficiency is a severe multisystem disorder with a wide range of clinical phenotypes most commonly affecting the nervous system. Caregiver reported data from the registry was consistent with the common disease characteristics documented in the literature and indicated that patients, particularly male patients, were well below the average for height and weight. Our characterization of known and novel *NGLY1* pathogenic variants facilitated calculation of a predicted incidence of NGLY1 Deficiency and may provide insight for *NGLY1* variant interpretation in the future.

In this dataset of the largest NGLY1 Deficiency cohort to date, the most commonly reported clinical features were global developmental delay/cognitive delay, absent speech, gait disturbance, hypotonia, alacrima, and severe constipation (all reported in > 86% of patients). It is possible that as more patients are described, the characteristic features of the disease reported by clinicians may evolve. The symptoms appear to be progressive, and regression of milestones was frequently reported, an important observation to convey when counseling newly diagnosed families, as well as for the design of therapeutic trials. For all patients older than 14 years old, caregivers reported regression of motor or cognitive function. Additionally, many patients were noted to be well below the norm for weight and height, which was consistent with reported feeding issues, need for g-tube placement, and physician reports of failure to thrive [2]. Shortened lifespan observed in this dataset was consistent with reports for NGLY1 Deficiency in at least 15% of cases [6, 26, 33].

Previous reports did not identify differences in disease phenotypes between males and females [3]. However, in our larger cohort, we observed that males had a significantly higher incidence of scoliosis, corneal scarring, abnormal EEG findings, and epilepsy compared to females. In addition, male growth was noted to be more severely affected. Females, on the other hand, had a significantly higher incidence of strabismus and constipation compared to males. The low Z-scores in height and weight suggest that failure to thrive is an important characteristic of this disease and that growth should be carefully monitored in patients with NGLY1 Deficiency.

Variants that contribute to NGLY1 Deficiency are universally thought to be due to loss of NGLY1 function. The majority are rare truncating variants that occur along the length of the protein. Pathogenic missense variants are enriched in the catalytic transglutaminase-like domain or in residues that are in close proximity to this domain. Missense variants should be interpreted cautiously if they fall outside of the catalytic domain (residues 275–391), especially if they are also not proximal to the catalytic domain (residues 190–274). Oligosaccharide screening by mass spectrometry could provide additional biochemical evidence for loss of NGLY1 function to aid variant interpretation [5]. Although we might anticipate that more severe mutations (e.g. truncating mutations) to lead to a more severe phenotype no such observation can be made in these data. This may be due to small numbers of affected subjects or that other factors (sex, environmental, other variants) have a strong modulating effect on phenotype that predominates over the effect of the causal mutation.

Most identified variants are in patients of European ancestry, likely due to ascertainment bias. However, several variants, while not common in the GSF cohort, have substantial frequencies in East Asian populations (Additional file 1: Table S3) and may indicate that this disorder could be more common in these populations than is currently recognized.

We calculated the incidence of NGLY1 Deficiency to be ~ 12 patients born per year in the United States. Historical data show that, for children born after 2007, GSF has identified approximately 1–2 new cases per birth year (data not shown), a smaller number than our theoretical calculations. Calculating the incidence of NGLY1 Deficiency in the general population is difficult due to the ultra-rare nature of the disease and the extremely long tail of vanishingly rare alleles that make up the majority of pathogenic alleles, which is an acknowledged limitation of our study. We suspect that the discrepancy between the observed and expected number of individuals with NGLY1 Deficiency, at least in part, implies that there are numerous, as yet undescribed, pathogenic alleles in the European population. Additionally, one allele (p.Gln631Serfs*7) is particularly common in the European portion of our cohort, yet is absent from gnomAD except in the Latino/Latina population; this may skew the calculated incidence by as much as 33%. Several other factors may also contribute to lower-than-expected numbers in the NGLY1 registry, including mortality prior to diagnosis, barriers to obtaining a molecular diagnosis, misdiagnosis, limited knowledge of and interest in connecting with a patient advocacy group, and increased use of reproductive genetic technologies. Data from animal models shows significantly reduced lifespan and increased rate of miscarriage [10], but additional work is needed to ascertain whether heterozygote parents also show increased miscarriage rates that would potentially reduce the true incidence of disease in humans.

In addition to assumptions made for the incidence calculation outlined above and previously reported, there are several limitations to this work. First, caregiver-reported data is subject to recall bias. As the survey was designed to be patient-centric, there are details of the clinical history that were not captured or may have been inaccurate. The lower-than-expected frequency of elevated liver transaminases and neuropathy reported in our cohort may be due to these limitations. Growth parameters reported may also have been impacted by caregiver recall bias and/or measurements performed at home.

## Conclusions

NGLY1 Deficiency is classified as an ultra-rare disease, but this assessment suggests it may be more common than is currently appreciated. At present, the condition is diagnosed via genetic testing. However, screening for the recently described NGLY1 Deficiency biomarker, GlcNAc-Asn, may help identify patients and increase the rate of diagnosis [5]. While the clinical features of NGLY1 Deficiency are relatively well described, explanations for the range in phenotypic severity, the progressive nature of the disease, observed differences between sexes for some clinical features, and potential for genotype–phenotype correlations are not well understood. Treatment options are presently limited to supportive care: however, a gene therapy is currently under development. Thus, it is increasingly important to identify affected families to participate in patient registries, natural history studies, and interventional studies.

## Supplementary Information

**Additional file 1**. Supplementary Tables 1–3 and Supplementary Figures 1–3.

## Data Availability

Data and materials may be supplied individually upon request through the Grace Science Foundation.
