# Supplementary material for: NGLY1 deficiency: estimated incidence, clinical features, and genotypic spectrum from the NGLY1 Registry
Source: Orphanet J Rare Dis. 2022 Dec 17;17:440. doi: 10.1186/s13023-022-02592-3 (PMC9759919; doi:10.1186/s13023-022-02592-3)
Supplement: Supplementary file 1 — Additional file 1. Supplementary Tables 1–3 and Supplementary Figures 1–3. [file 13023_2022_2592_MOESM1_ESM.docx]

**Supplemental Table 1.** NGLY1 Patients in GSF Cohort and Literature

| **Age(y)** | **Sex** | **Variant 1** | **Variant 2** | **Sibs** | **Phenotypic data** | **GSF cohort and/or previous publication (s)** | **Previously unreported variant** |
| --- | --- | --- | --- | --- | --- | --- | --- |
| 6 | F | p.Cys283Trp | p.Arg401* |  | Yes | Levy et al (2022) | No |
| 4 | F | c.1150-1 G>C | p.Arg401* |  | Yes | Levy et al (2022) | No |
| 9 | F | p.Gln631Serfs*7 | c.1789+5 G>A |  | Yes | This cohort | Yes |
| d.17 | M | Not available^1^ | Not available^1^ |  | Yes | This cohort | No |
| 18 | F | p.Arg401* | p.Glu356Gly |  | Yes | Panneman et al., (2020) | No |
| 12 | M | p.Gln191* | p.Gln191* |  | Yes | Levy et al (2022) | No |
| 8 | F | c.1150-1 G>C | p.Arg401* |  | Yes | Levy et al (2022) | No |
| 5 | M | c.1789+1G>T | p.Arg308Trp |  | Yes | Levy et al (2022) | No |
| 28 | F | p.Arg458Lysfs*14 | p.Arg458Lysfs*14 |  | Yes | Enns et al (2014); Lam et al (2017); Levy et al (2022) | No |
| 8 | F | p.Arg469* | p.Arg469* |  | Yes | Chang et al (2019); Levy et al (2022) | No |
| 10 | M | p.Arg401* | p.Arg401* |  |  | Enns et al (2014) ; Lam et al (2017) ; Levy et al (2022) | No |
| 11 | F | p.Arg401* | p.Arg401* |  |  | Enns et al (2014);Lam et al (2017) | No |
| 25 | F | p.Arg401* | p.Arg401* | Yes |  | Enns et al (2014);Lam et al (2017) | No |
| d.22 | F | p.Arg401* | p.Arg401* |  |  | This cohort | No |
| 9 | M | p.Leu318Pro | p.Arg390Pro |  |  | Lam et al (2017) | No |
| 5 | M | p.Gln631Serfs*7 | p.Gln631Serfs*7 |  |  | This cohort | No |
| 14 | F | p.Arg469* | p.Asp597Valfs*20 |  | Yes | Levy et al (2022) | No |
| 9 | M | p.Gln631Serfs*7 | p.Asn178Glnfs*9 |  | Yes | Rios-Flores et al (2020); Levy et al (2022) | No |
| 19 | F | p.Asn511Lysfs*51 | p.Asn511Lysfs*51 |  |  | Caglayan et al (2015) | No |
| 9 | M | p.Arg401* | p.Arg401* |  |  | This cohort | No |
| 6 | M | del.intron3-exon4 | p.Arg401* |  | Yes | Levy et al (2022) | No |
| 40 | F | p.Asn511Lysfs*51 | p.Asn511Lysfs*51 |  |  | This cohort | No |
| 29 | F | p.Asn511Lysfs*51 | p.Asn511Lysfs*51 | Yes |  | This cohort | No |
| d.14 | M | p.Arg401* | p.Arg401* |  |  | This cohort | No |
| 10 | M | p.Arg469* | p.Cys355Tyr |  |  | Levy et al (2022) | No |
| 4 | M | p.Arg401* | p. Asn511Lysfs*51 |  |  | Levy et al (2022) | No |
| 18 | F | p.Asn415Metfs*23 | c.658+1 G>A |  | Yes | Levy et al. (2022) | No |
| d.22 | M | p.Ser116* | c.881+5 G>T |  | Yes | Lam et al (2014); Heeley et al (2018); Levy et al (2022) | No |
| d.22 | F | p.Arg401* | p.Arg401* |  | Yes | Lam et al (2017); Levy et al (2022) | No |
| 17 | M | p.G310G splice error | p.Gln208* |  | Yes | Lam et al (2017); Levy et al (2022) | No |
| 14 | F | p.G310G splice error | p.Gln208* | Yes | Yes | Lam et al (2017); Levy et al (2022) | No |
| 12 | F | p.Glu311Lys | p.Trp244Arg |  | Yes | Lam et al (2017); Levy et al (2022) | No |
| 12 | M | p.His494Leufs*14 | p.Glu311Lys |  | Yes | This cohort | No |
| d.15 | M | p.Arg401* | del. exon 1-3 |  | Yes | Levy et al (2022) | No |
| d.12 | M | p.Gln631Serfs*7 | p.Arg401* |  | Yes | Need et al (2012);Enns et al (2014);Lam et al (2017);Levy et al (2022) | No |
| 7 | F | p.Glu432* | p.Glu432* |  | Yes | This cohort | No |
| 18 | M | p.Ser169* | p.Arg383* |  | Yes | Levy et al (2022) | No |
| 9 | M | p.Ser169* | p.Arg383* | Yes | Yes | Levy et al (2022) | No |
| 9 | M | p.Arg401* | p.Arg308Trp |  | Yes | This cohort | Yes |
| 17 | M | p.Arg401* | p.Arg401* |  | Yes | This cohort | No |
| 17 | M | p.Gln631Serfs*7 | p.Arg321* |  | Yes | Levy et al (2022) | No |
| 9 | F | p.Ser267Valfs*5 | p.Ser267Valfs*5 |  | Yes | This cohort | Yes |
| 6 | M | p.Ser267Valfs*5 | p.Ser267Valfs*5 | Yes | Yes | This cohort | No |
| 11 | M | p.Arg411* | p.Arg411* |  | Yes | This cohort | No |
| 12 | F | p.Arg402del | p.Arg542* |  | Yes | Enns et al (2014); Levy et al (2022) | No |
| 20 | M | p.Gln631Serfs*7 | p.Gln631Serfs*7 |  | Yes | This cohort | No |
| 12 | M | p.Trp535* | p.Leu637* |  | Yes | Lam et al (2017); Levy et al (2022) | No |
| 11 | F | p.Gln631Serfs*7 | p.Gln631Serfs*7 |  | Yes | This cohort | No |
| d.5 | M | p.Arg411* | p.Arg411* |  |  | This cohort | No |
| 5 | F | p.Arg401* | p.Ser607Phefs*5 |  |  | Levy et al (2022) | No |
| 8 | F | p.Trp369* | p.Arg469* |  |  | Levy et al (2022) | No |
| 17 | F | p.Tyr497* | p.Tyr497* |  |  | This cohort | Yes |
| 3 | F | p.Arg401* | del. entire coding sequence |  | Yes | Levy et al (2022) | No |
| 3 | F | p.Arg401* | del. entire coding sequence | Yes | Yes | Levy et al (2022) | No |
| 7 | F | p.Arg308Trp | p.Tyr550Serfs*7 |  |  | This cohort | Yes |
| 2 | M | p.Arg401* | del. exon 1 |  |  | This cohort | No |
| 4 | M | p.Arg401* | c.250G>T p.Glu84* |  |  | Lipiński,Bogdańska, et al (2020) | No |
| 3 | M | p.Arg401* | p.Arg401* |  |  | This cohort | No |
| 3 | M | p.Arg401* | p.Arg401* |  |  | This cohort | No |
| 15 | M | p.Cys484fs | p.Cys484fs |  |  | This cohort | Yes |
| 3 | M | p.Arg401* | p.Thr630Profs*4 |  |  | This cohort | Yes |
| 6 | F | p. Asn511Lysfs*51 | not identified^2^ |  |  | This cohort | No |
| 3 | F | p.Glu326_Ala327delinsAsp | p.Arg328Cys |  |  | This cohort | No |
| 9 | M | c.1789+1G>A | p.Cys355Arg |  |  | Lipiński,Cielecka-Kuszyk, et al (2020) | No |
| 8 | M | p.Arg321* | p.Tyr391His |  |  | This cohort | Yes |
| 2 | M | p.Arg401* | p.Arg401* |  |  | This cohort | No |
| 2 | F | p.Arg401* | p.Gln631Serfs*7 |  |  | This cohort | No |
| 23 | M | p.Asn511Lysfs*51 | p.Tyr342Cys |  |  | This cohort | No |
| 3 | M | p.Trp389* | p.Gln176* |  |  | This cohort | Yes x2 |
| 11m | F | p.Arg401* | p.Arg401* |  |  | This cohort | No |
| 1 | M | p.R390* | p.P135Lfs*11 |  |  | This cohort | Yes |
| d.18 | F | p.Try539Glyfs*19 | p.Leu618* |  |  | Haijes et al (2019) | No |
| 3 | M | p.Arg469* | p.Arg469* |  |  | This cohort | No |
| 14 | M | p.Arg469* | p.Arg469* |  |  | This cohort | No |
| d.5 | M | p.Arg401* | p.Arg401* | Yes |  | Enns et al (2014) | No |
| d.9 m | F | p.Arg401* | p.Arg401* |  |  | Enns et al (2014) | No |
| d.16 | M | p.Asn511Lysfs*51 | p.Asn511Lysfs*51 |  |  | Caglayan et al (2015) | No |
|  | unk. | p.Arg401* | p.Arg401* |  |  | Bosch et al (2016) | No |
| 8 | F | p.Q613fs | p.Q613fs |  |  | van Kuelen et al (2019); Panneman (2020) | No |
| 18 | M | c.247-2A>G | c.247-2A>G | Yes |  | Haijes et al (2019) | No |
| 26 | F | c.247-2A>G | c.247-2A>G |  |  | Haijes et al (2019) | No |
| 11 | M | p.Arg586* | p.Arg586* | Yes |  | Haijes et al (2019) | No |
| 6 | F | p.Arg586* | p.Arg586* |  |  | Haijes et al (2019) | No |
| 17 m | M | p.Tyr342Cys | p.Arg411* |  |  | Abuduxikuer et al (2020) | No |
| 5 | F | p.Tyr342Cys | p.Arg411* |  |  | Abuduxikuer et al (2020) | No |
| 25 m | F | p.Tyr342Cys | p.Arg411* |  |  | Abuduxikuer et al (2020) | No |
| 8 m | F | p.Ser546Phefs* | c.1003+3A>G | Yes |  | Abuduxikuer et al (2020) | No |
| 4 | F | p.Ser546Phefs* | c.1003+3A>G |  |  | Abuduxikuer et al (2020) | No |
| 22 m | M | p.Arg328Cys | p.Arg328Cys |  |  | Abuduxikuer et al (2020) | No |
| unk. | unk. | p.Cys283Trp | p.Arg401* |  |  | Panneman et al (2020) | No |
| unk. | unk. | p.Arg401* | p.Arg401* |  |  | Panneman et al (2020) | No |
| 8 | M | p.Gln631Serfs*7 | p.Gln631Serfs*7 |  |  | Lipari Pinto et al (2020) | No |
| 10 m | F | p.Asp386Tyr | p.R390* | Yes |  | Ge et al (2020) | No |
| 30 | M | p.Typ236Cys | p.Typ236Cys | Yes |  | Kariminijad et al (2021) | No |
| 34 | M | p.Typ236Cys | p.Typ236Cys |  |  | Kariminijad et al (2021) | No |
| 35 | F | p.Typ236Cys | p.Typ236Cys |  |  | Kariminijad et al (2021) | No |
| 14 | F | p.Arg390Gln | p.Arg390Gln | Yes |  | Kariminijad et al (2021) | No |
| 29 | F | p.Arg390Gln | p.Arg390Gln |  |  | Kariminijad et al (2021) | No |
| d.6.5 | F | p.Arg328Cys | p.Arg328Cys |  |  | Dabaj et al (2021) | No |
| 6 | M | p.Trp244* | p.Trp244* |  |  | Zidoune et al (2021) | No |
| d.6 m | F | c.1294G>T | c.1294G>T | Yes |  | Kalfon et al (2022) | No |
| d.3 | M | c.1294G>T | c.1294G>T |  |  | Kalfon et al (2022) | No |
| d.12^3^ | F | c.1294G>T | c.1294G>T |  |  | Kalfon et al (2022) | No |

^1. Genetic testing results not available but confirmed via clinician report^

^2. Diagnosis based upon phenotype, biomarker, and clinician opinion^

^3. Patient reported as living, but CRS learned patient deceased by personal communication between CRS and author^

**Supplemental Table 2.** Descriptive Statistics of Growth

|  | | **Height** | | | **Weight** | |
| --- | --- | --- | --- | --- | --- | --- |
|  |  | Z-score | %ile | Z-score | | %ile |
| **M & F (2-20y)** | Median | -2.43 | 0.8 | -1.64 | | 5 |
|  | Min | -7.4 | 0 | -4.78 | | 0 |
|  | Max | -0.14 | 44.4 | 0.84 | | 79.9 |
|  | Mean | -2.6 | 10.0 | -1.4 | | 21.4 |
|  | StdDev | 1.8 | 20.8 | 1.7 | | 29.7 |
| **F (2-20y)** | Median | -2.4 | 0.8 | -1.3 | | 9.2 |
|  | Min | -4.2 | 0.0 | -2.8 | | 0.3 |
|  | Max | -0.1 | 44.4 | 0.8 | | 79.9 |
|  | Mean | -2.3 | 12.4 | -1.1 | | 26.2 |
|  | StdDev | 1.6 | 17.8 | 1.2 | | 29.9 |
| **M (2-20y)** | Median | -2.6 | 0.6 | -1.9 | | 3.4 |
|  | Min | -7.4 | 0.0 | -4.8 | | 0.0 |
|  | Max | 1.8 | 96.4 | 3.7 | | 100 |
|  | Mean | -2.9 | 7.9 | -1.7 | | 18.3 |
|  | StdDev | 2.0 | 24.0 | 2.1 | | 29.2 |

**Supplemental Table 2.** Height and Weight Summary Statistics for participants aged 2-20 years for whom height and weight data was available by caregiver report compared to CDC growth curves.

**Supplemental Figure 1.** NGLY1 structure of catalytic domain


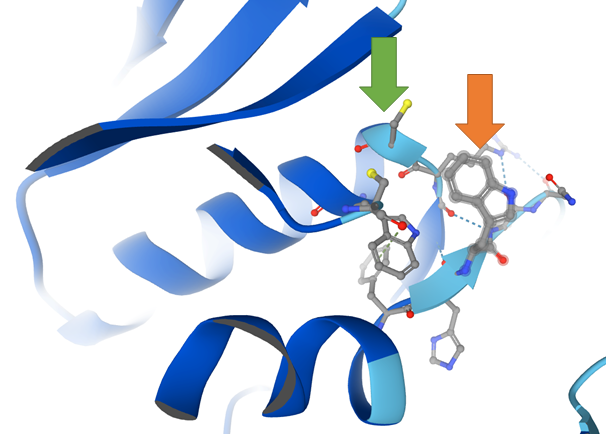


**Supplemental Figure 1.** Structure of NGLY1 alpha-helix residues 233-244 and 309-323: Tyr244Arg** (orange arrow) interacts closely with the transglutaminase domain residue Cys309 (green arrow) and may indicate mechanism of pathogenicity.

**Supplemental Figure 2.** Allele frequency Distribution.
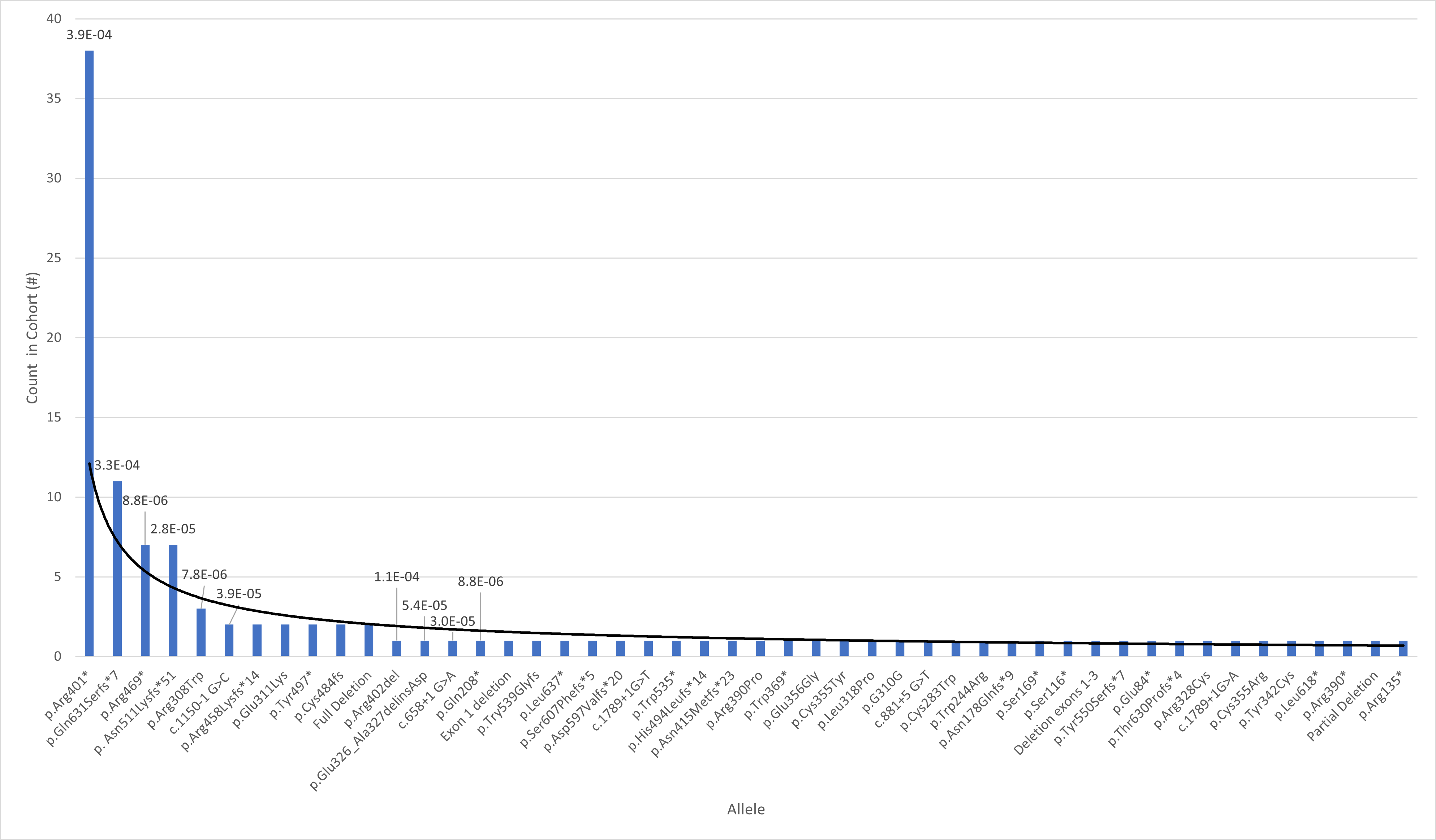


**Supplemental Figure 2.** Allele Frequency Distribution. Variant occurrence in cohort listed in descending order with (non-zero) minor allele frequency to show long tail of rare pathogenic alleles in European population.

**Supplemental Figure 3.** Patients Identified Over Time


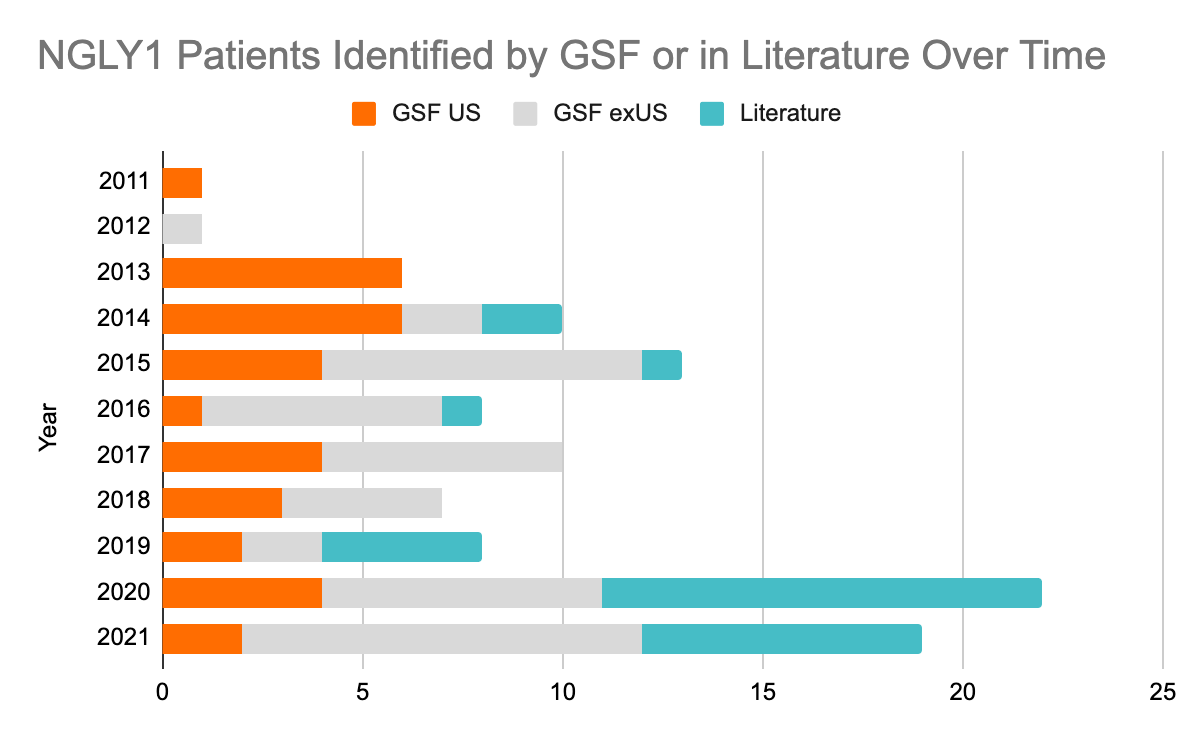


**Supplemental Figure 3.** Annual number of NGLY1 Deficiency patients identified by Grace Science Foundation or reported in the literature for years since 2011. Patients identified by Grace Science Foundation in the US colored orange, patients identified by Grace Science Foundation outside the US colored gray, patients reported in the literature (not in contact with the Foundation) colored in turquoise.

| **Ch.** | **Position** | **HGVS  Consequence** | **Allele Count (Total)** | **Allele Frequency (Total)** | **East Asian** | **African/African American** | **European (non-Finnish)** | **European (Finish)** | **Latino/Admixed American** | **Ashkenazi Jewish** | **South Asian** | **Other** |
| --- | --- | --- | --- | --- | --- | --- | --- | --- | --- | --- | --- | --- |
| 3 | 25761024 | p.Gln631SerfsTer7 | 2 | 7.96E-06 | 0.00E+00 | 0.00E+00 | 0.00E+00 | 0.00E+00 | 0.00E+00 | 0.00E+00 | 0.00E+00 | 3.26E-04 |
| 3 | 25761500 | c.1789+5G>A | 1 | 4.02E-06 | 0.00E+00 | 0.00E+00 | 8.88E-06 | 0.00E+00 | 0.00E+00 | 0.00E+00 | 0.00E+00 | 0.00E+00 |
| 3 | 25761670 | p.Arg542Ter | 2 | 7.98E-06 | 1.09E-04 | 0.00E+00 | 0.00E+00 | 0.00E+00 | 0.00E+00 | 0.00E+00 | 0.00E+00 | 0.00E+00 |
| 3 | 25770698 | p.Asn511LysfsTer51 | 4 | 1.70E-05 | 0.00E+00 | 0.00E+00 | 2.77E-05 | 0.00E+00 | 0.00E+00 | 0.00E+00 | 3.50E-05 | 0.00E+00 |
| 3 | 25773830 | p.Arg469Ter | 5 | 1.99E-05 | 1.63E-04 | 0.00E+00 | 8.81E-06 | 0.00E+00 | 2.90E-05 | 0.00E+00 | 0.00E+00 | 0.00E+00 |
| 3 | 25775392 | p.Arg411Ter | 7 | 2.79E-05 | 2.18E-04 | 1.23E-04 | 8.80E-06 | 0.00E+00 | 0.00E+00 | 0.00E+00 | 0.00E+00 | 0.00E+00 |
| 3 | 25775415 | p.Arg402del | 2 | 7.96E-06 | 1.09E-04 | 0.00E+00 | 0.00E+00 | 0.00E+00 | 0.00E+00 | 0.00E+00 | 0.00E+00 | 0.00E+00 |
| 3 | 25775422 | p.Arg401Ter | 52 | 1.84E-04 | 0.00E+00 | 4.01E-05 | 3.87E-04 | 0.00E+00 | 0.00E+00 | 0.00E+00 | 0.00E+00 | 1.39E-04 |
| 3 | 25775474 | c.1150-1G>C | 5 | 1.77E-05 | 0.00E+00 | 0.00E+00 | 3.88E-05 | 0.00E+00 | 0.00E+00 | 0.00E+00 | 0.00E+00 | 0.00E+00 |
| 3 | 25777619 | p.Tyr342Cys | 3 | 1.20E-05 | 0.00E+00 | 0.00E+00 | 1.77E-05 | 0.00E+00 | 0.00E+00 | 0.00E+00 | 3.28E-05 | 0.00E+00 |
| 3 | 25778847 | p.Glu326_Ala327delinsAsp | 7 | 2.49E-05 | 0.00E+00 | 0.00E+00 | 5.44E-05 | 0.00E+00 | 0.00E+00 | 0.00E+00 | 0.00E+00 | 0.00E+00 |
| 3 | 25778867 | p.Arg321Ter | 2 | 7.97E-06 | 0.00E+00 | 0.00E+00 | 8.81E-06 | 0.00E+00 | 0.00E+00 | 0.00E+00 | 3.27E-05 | 0.00E+00 |
| 3 | 25778906 | p.Arg308Trp | 1 | 3.19E-05 | 0.00E+00 | 4.01E-05 | 7.77E-06 | 0.00E+00 | 0.00E+00 | 0.00E+00 | 0.00E+00 | 0.00E+00 |
| 3 | 25781075 | p.Cys286PhefsTer5 | 1 | 3.18E-05 | 0.00E+00 | 1.15E-04 | 0.00E+00 | 0.00E+00 | 0.00E+00 | 0.00E+00 | 0.00E+00 | 0.00E+00 |
| 3 | 25781078 | p.Arg291Ter | 1 | 3.99E-06 | 5.44E-05 | 0.00E+00 | 0.00E+00 | 0.00E+00 | 0.00E+00 | 0.00E+00 | 0.00E+00 | 0.00E+00 |
| 3 | 25792588 | c.658+1G>A | 2 | 8.10E-06 | 0.00E+00 | 0.00E+00 | 0.00E+00 | 0.00E+00 | 2.99E-05 | 0.00E+00 | 0.00E+00 | 1.67E-04 |
| 3 | 25792625 | p.Gln208Ter | 1 | 4.00E-06 | 0.00E+00 | 0.00E+00 | 8.83E-06 | 0.00E+00 | 0.00E+00 | 0.00E+00 | 0.00E+00 | 0.00E+00 |

**Supplemental Table 3.** Allele frequencies from gnomAD

**Supplemental Table 3.** NGLY1 variants from GSF with available minor allele frequency in gnomAD.
